# Supplementary material for: Causal Relationship between Aging and Anorexia Nervosa: A White-Matter-Microstructure-Mediated Mendelian Randomization Analysis
Source: Biomedicines. 2024 Aug 16;12(8):1874. doi: 10.3390/biomedicines12081874 (PMC11351342; doi:10.3390/biomedicines12081874)
Supplement: Supplementary file 1 [file biomedicines-12-01874-s001.zip › Supplementary Figure/Figure S4/Singe SNP plot of MR analysis for TL to ubm-b-2025.pdf]

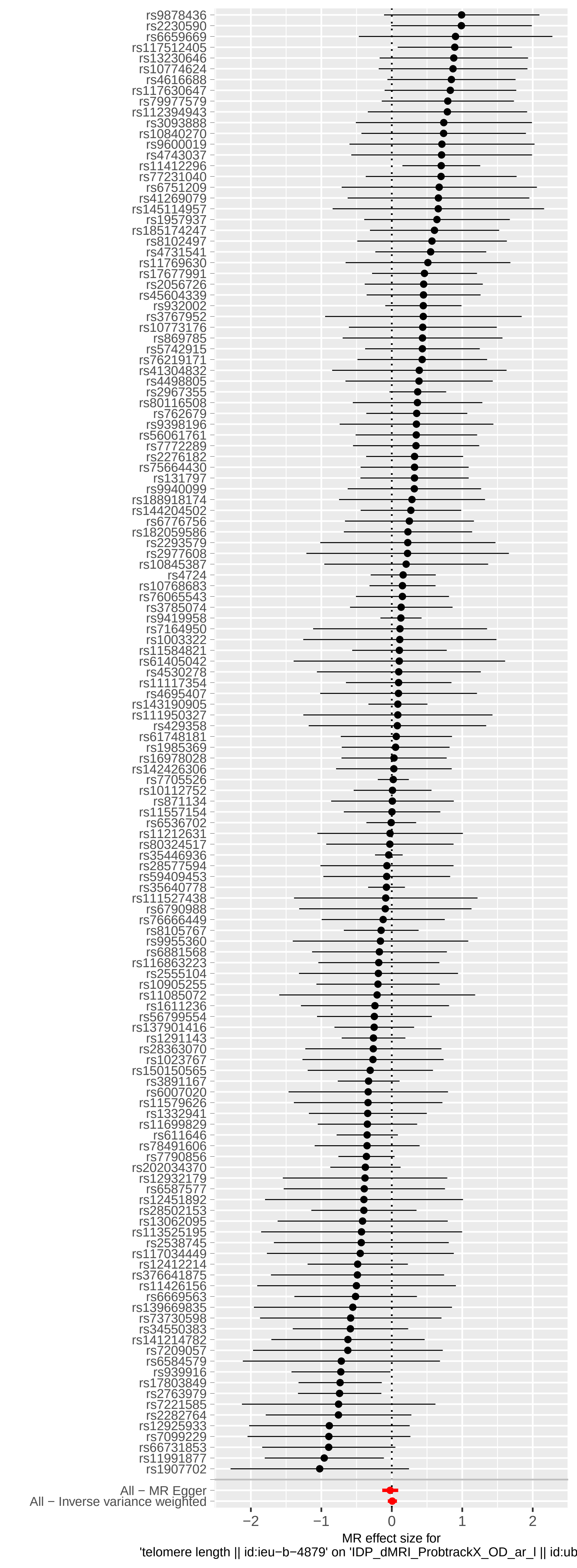
rs9878436
rs2230590
rs6659669
rs117512405
rs13230646
rs10774624
rs4616688
rs117630647
rs79977579
rs112394943
rs3093888
rs10840270
rs9600019
rs4743037
rs11412296
rs77231040
rs6751209
rs41269079
rs145114957
rs1957937
rs185174247
rs8102497
rs4731541
rs11769630
rs17677991
rs2056726
rs45604339
rs932002
rs3767952
rs10773176
rs869785
rs5742915
rs76219171
rs41304832
rs4498805
rs2967355
rs80116508
rs762679
rs9398196
rs56061761
rs7772289
rs2276182
rs75664430
rs131797
rs9940099
rs188918174
rs144204502
rs6776756
rs182059586
rs2293579
rs2977608
rs10845387
rs4724
rs10768683
rs76065543
rs3785074
rs9419958
rs7164950
rs1003322
rs11584821
rs61405042
rs4530278
rs11117354
rs4695407
rs143190905
rs111950327
rs429358
rs61748181
rs1985369
rs16978028
rs142426306
rs7705526
rs10112752
rs871134
rs11557154
rs6536702
rs11212631
rs80324517
rs35446936
rs28577594
rs59409453
rs35640778
rs111527438
rs6790988
rs76666449
rs8105767
rs9955360
rs6881568
rs116863223
rs2555104
rs10905255
rs11085072
rs1611236
rs56799554
rs137901416
rs1291143
rs28363070
rs1023767
rs150150565
rs3891167
rs6007020
rs11579626
rs1332941
rs11699829
rs611646
rs78491606
rs7790856
rs202034370
rs12932179
rs6587577
rs12451892
rs28502153
rs13062095
rs113525195
rs2538745
rs117034449
rs12412214
rs376641875
rs11426156
rs6669563
rs139669835
rs73730598
rs34550383
rs141214782
rs7209057
rs6584579
rs939916
rs17803849
rs2763979
rs7221585
rs2282764
rs12925933
rs7099229
rs66731853
rs11991877
rs1907702
All − MR Egger
All − Inverse variance weighted
−2
−1
0
1
2
MR effect size for
'telomere length || id:ieu−b−4879' on 'IDP_dMRI_ProbtrackX_OD_ar_l || id:ub
